# Mitochondrial targeted catalase improves muscle strength following arteriovenous fistula creation in mice with chronic kidney disease

**DOI:** 10.1038/s41598-024-58805-1

**Published:** 2024-04-09

**Authors:** Kyoungrae Kim, Brian Fazzone, Tomas A. Cort, Eric M. Kunz, Samuel Alvarez, Jack Moerschel, Victoria R. Palzkill, Gengfu Dong, Erik M. Anderson, Kerri A. O’Malley, Scott A. Berceli, Terence E. Ryan, Salvatore T. Scali

**Affiliations:** 1 Division of Vascular Surgery and Endovascular Therapy, University of Florida, P.O. Box 100128, Gainesville, FL 32610 USA; 2 Department of Applied Physiology and Kinesiology, University of Florida, 1864 Stadium Rd, Gainesville, FL 32611 USA; 3 Center for Exercise Science, University of Florida, Gainesville, FL USA; 4 Malcom Randall Veteran Affairs Medical Center, Gainesville, FL USA

**Keywords:** Arteriovenous fistula, Dialysis, End-stage kidney disease, Mitochondria, Haemodialysis, Experimental models of disease, Preclinical research

## Abstract

Hand dysfunction is a common observation after arteriovenous fistula (AVF) creation for hemodialysis access and has a variable clinical phenotype; however, the underlying mechanism responsible is unclear. Grip strength changes are a common metric used to assess AVF-associated hand disability but has previously been found to poorly correlate with the hemodynamic perturbations post-AVF placement implicating other tissue-level factors as drivers of hand outcomes. In this study, we sought to test if expression of a mitochondrial targeted catalase (mCAT) in skeletal muscle could reduce AVF-related limb dysfunction in mice with chronic kidney disease (CKD). Male and female C57BL/6J mice were fed an adenine-supplemented diet to induce CKD prior to placement of an AVF in the iliac vascular bundle. Adeno-associated virus was used to drive expression of either a green fluorescent protein (control) or mCAT using the muscle-specific human skeletal actin (HSA) gene promoter prior to AVF creation. As expected, the muscle-specific AAV-HSA-mCAT treatment did not impact blood urea nitrogen levels (*P* = 0.72), body weight (*P* = 0.84), or central hemodynamics including infrarenal aorta and inferior vena cava diameters (*P* > 0.18) or velocities (*P* > 0.38). Hindlimb perfusion recovery and muscle capillary densities were also unaffected by AAV-HSA-mCAT treatment. In contrast to muscle mass and myofiber size which were not different between groups, both absolute and specific muscle contractile forces measured via a nerve-mediated in-situ preparation were significantly greater in AAV-HSA-mCAT treated mice (*P* = 0.0012 and *P* = 0.0002). Morphological analysis of the post-synaptic neuromuscular junction uncovered greater acetylcholine receptor cluster areas (*P* = 0.0094) and lower fragmentation (*P* = 0.0010) in AAV-HSA-mCAT treated mice. Muscle mitochondrial oxidative phosphorylation was not different between groups, but AAV-HSA-mCAT treated mice had lower succinate-fueled mitochondrial hydrogen peroxide emission compared to AAV-HSA-GFP mice (*P* < 0.001). In summary, muscle-specific scavenging of mitochondrial hydrogen peroxide significantly improves neuromotor function in mice with CKD following AVF creation.

## Introduction

End stage kidney disease (ESKD) is the most severe manifestation of chronic kidney disease (CKD) and the number of patients suffering from debilitating advanced renal disease is expected to significantly increase over the coming decades^1^. Unlike CKD patients, ESKD patients require renal replacement therapy to sustain life^2^. Hemodialysis is the most prevalent renal replacement therapy which necessitates establishing a durable hemoaccess through surgical construction of either an arteriovenous fistula (AVF) or graft^3^. In both cases, achieving a patent access that can be easily cannulated without stenosis, thrombosis, or infection are critical characteristics of a reliable vascular access to facilitate hemodialysis. Unfortunately, approximately 30–60% of patients experience dialysis access-related hand dysfunction (ARHD) which can manifest with a spectrum of symptoms ranging from subtle paresthesia, neuromotor discoordination, and/or weakness. Moreover, in rare cases, ARHD patients can experience severe mono-paresis and some even develop digital gangrene^4^. Undoubtedly, the primary goal of AVF creation is generation of a dependable hemoaccess but symptoms of ARHD can have a significant negative impact the patient’s quality of life. Importantly, the underlying mechanisms responsible for ARHD and a unifying pathobiological explanation for why there is wide variation in the observed clinical manifestations is unknown. Hemodynamic changes caused by AVF creation have been implicated; however, recent work has shown a lack of association between hand perfusion and ARHD^4–6^. This gap in knowledge is a barrier toward the development of adjuvant therapies and/or refinement of the surgical processes that could attenuate the risk and/or severity of ARHD and improve clinical outcomes for patients undergoing dialysis-access procedures.

Considering that hemodynamic changes post-AVF creation do not correlate with metrics of ARHD^4^, it is plausible that local maladaptive changes to the skeletal muscle distal to the AVF may be involved in the etiology of ARHD. In support of this hypothesis, there is a growing body of literature demonstrating that CKD alone has profound negative effects on skeletal muscle^7–10^, including atrophy and muscle weakness which have been associated with elevated levels of oxidative stress and impaired mitochondrial function^9–17^. On this basis, it is reasonable to hypothesize that the hemodynamic challenge imposed by AVF creation may have additive or synergistic pathological effects to the skeletal muscle distal to the anastomosis thereby promoting symptoms of ARHD. To begin to explore whether skeletal muscle pathology contributes to ARHD following AVF creation, our group developed a novel mouse model in which an AVF can be created in the iliac vascular bundle allowing examination of limb pathophysiology^18,19^ which is not possible using alternative pre-clinical models using aortic or carotid vessels^20–22^. In this murine iliac AVF model, mice have been shown to exhibit hindlimb weakness, gait abnormalities, and muscle mitochondrial abnormalities^18,23,24^. Oxidative stress and mitochondrial dysfunction have been frequently reported in the skeletal muscle of rodents and human patients with CKD^10–12,16,25–28^. Independent of CKD, hemodynamic challenges in skeletal muscle have also been shown to elevate oxidative stress^29–31^. In the present study, we tested the hypothesis that treatment with a mitochondrial targeted catalase in skeletal muscle prior to the creation of an AVF could mitigate limb dysfunction post-operatively.

## Methods

### Animals and CKD induction

Male and female 10-week-old C57BL/6J mice were purchased from Jackson Laboratory and housed in the animal facility in which temperature (22 ± 1 °C), humidity (~ 50%), and light (12 h light and 12 h dark cycle) are controlled. One week following arrival, all mice were switched to a 20% casein-based chow diet. After 1 week on casein diet, all mice were fed a 0.2% adenine-supplemented casein chow to induce chronic kidney disease prior to the AVF surgery and remained on the adenine diet throughout the study as previously described^18,23,24,32–35^. All animals were provided both food and water ad libitum for the duration of the experiment. Three days prior to undergoing AVF surgery, approximately 20 µl of whole blood were collected via a one-millimeter tail snip to measure blood urea nitrogen via a commercially available colorimetric assay kit (K024, Arbor Assays). All animal experiments adhered to the Guide for the Care and Use of Laboratory Animals from Institute for Laboratory Animal Research (National Academy Press, National Research Council, Washington, DC, 2011) and any updates. The institutional animal care and use committee of the University of Florida and Malcom Randall Veterans Affairs Medical Center approved all procedures. This study was conducted and reported in accordance with the ARRIVE Guidelines (https://arriveguidelines.org).

### Plasmid constructs and adeno-associated virus generation and delivery

The catalase (CAT) coding sequence was PCR amplified from cDNA generated from a liver obtained from a C57BL/6J mouse using CloneAmp HiFI PCR premix (Takara Bio). The primers used to amplify the murine CAT coding sequence were generated to incorporate a N-terminus 32-amino acid mitochondrial targeting sequence from the human ornithine transcarbamoylase enzyme as previously described^36^. To accomplish muscle cell-specific expression of the mitochondrial-targeted catalase (mCAT), the human ACTA1 promoter (1541-bp proximal to transcription start site) was PCR amplified from human-genomic DNA isolated from a donor muscle biopsy. The HSA promoter and mCAT coding sequence were inserted into a promoterless AAV cloning vector (CellBio) using In-Fusion cloning (Takara Bio). A plasmid containing a green fluorescent protein (ZsGreen1, GFP) driven by the HSA promoter was used as a control group. AAV’s were packaged using the AAV9 serotype and obtained from Vector Biolabs (Malvern, PA). Two weeks prior to the AVF surgery, AAV’s were injected intramuscularly to the left tibialis anterior (TA, 50 µl injection volume) and extensor digitorum longus (EDL, 20 µl injection volume) muscles and gastrocnemius/plantaris/soleus (80 µl injection volume) muscle complex of surgical limb at 5 × 10^11^ vg/mouse. Mice were randomly assigned to the AAV treatment groups, and all experimenters were blinded until analysis was completed.

### Animal model of hemodialysis access-related hand dysfunction

Three weeks after the 0.2% adenine diet application, animals underwent microsurgery to create an AVF on the left common iliac vessels as previously described^18,19,23^. Briefly, mice were anesthetized with initial isoflurane anesthesia, buprenorphine HCl (0.1 mg/kg) was administered subcutaneously, and the abdominal hair was removed using a pen-trimer. Positioned supine on a warming pad, the surgical field was disinfected with alternating chlorhexidine and alcohol wipes and a midline laparotomy from the pubis symphysis to the sternal margin was made. Next, the intraperitoneal components including small and large bowel, seminal vesicles, uterine horns, and ureters were gently moved and covered by a saline-soaked non-woven sponge to expose the target vessels. Heparinized saline (0.2 IU/g) was injected via the inferior vena cava to prevent coagulation/thrombosis and improve AVF patency outcomes. Small vessel branches that can cause bleeding were ligated using low-temperature cautery and the left common iliac artery and vein were carefully dissected from the underlying retroperitoneal musculature using straight and angled forceps. Two 4-0 silk sutures were placed at the proximal and distal ends of the iliac artery-iliac vein bundle and used to control the vasculature for AVF creation. Following the longitudinal venotomy (~ 1 mm), a 10-0 nylon imbricating suture was used to gently displace the posterior iliac vein and juxtaposed anterior iliac artery wall. Once this was completed, an approximately 0.8-9 mm × 0.3 mm elliptical incision was made into the conjoined poster iliac vein/anterior iliac artery wall to establish the fistula connection using Vannas spring scissors, followed by 0.9% saline wash. The anterior wall venotomy incision was repaired using two or three interrupted 10-0 nylon sutures followed by placing the vascular bundle into its normal anatomical position. Subsequently, the two 4-0 silk suture vessel loops were loosened carefully and the site of the arteriovenous anastomosis, as well as the surrounding tissues were gently blotted by saline-soaked pointed cotton swabs to restore blood flow. AVF patency was confirmed by visualization of pulsatile, bright red oxygenated blood entering the iliac vein and mixing with dark venous blood returning from the hindlimb. The mid-line celiotomy and skin incisions were closed using absorbable sutures. All mice were treated with buprenorphine (0.1 mg/kg) post-operatively for 48 h as analgesia. Mice were monitored daily post-operatively and moisturized food was given in warmed recovery cages for three days post-operatively.

### Assessment of fistula patency and systemic hemodynamics

The patency of AVF and hemodynamics of infrarenal aorta and inferior vena cava were assessed using a high-frequency duplex ultrasound system (30–50 MHz, Vevo 2100, VisualSonics, Inc. Toronto, Ontario, Canada) 4 days prior to the euthanasia as previously described^18,23,37^. After induction of general anesthesia via isoflurane, mice were placed on a prewarmed 3D positioning platform in a supine position and their extremities were fixed with tape. AVF patency was ascertained using color and pulse-wave Doppler ultrasound where the increased peak systolic velocity with mixed arteriovenous blood flow existed in the juxta-anastomotic position. The diameter and peak systolic velocity of the infrarenal aorta and inferior vena cava were assessed using B-mode imaging and pulse-wave Doppler ultrasound at an insonation angle of 60°.

### Assessment of hindlimb perfusion

Post-operative blood perfusion to the TA muscle and ventral paw was assessed using a laser Doppler flowmeter (moorVMS-LDF, Moor Instruments) immediately after the AVF creation and post-operatively on days 3 and 13 as described previously^23^. The average perfusion flux (~ 10 s) was determined, and perfusion recovery was calculated as a percentage of AVF limb (left) relative to the contralateral non-surgical limb (right).

#### Immunoblotting

To validate mitochondrial catalase overexpression, isolated TA muscles were lysed in CelLytic M (Millipore-Sigma, Cat. No. C2978) and protein concentrations were determined by bicinchoninic acid protein assay (ThermoFisher Scientific, Cat No. A53225). Lysates were mixed with Laemmli sample buffer (Bio-Rad, Cat. No. 1610737) supplemented with beta-mercaptoethanol and proteins were separated using SDS-PAGE gels (Bio-Rad, Cat. No. 4561043) and subsequently transferred to polyvinylidene fluoride (PVDF) membranes. PVDF membranes were then blocked for 90 min in blocking buffer (Licor, Cat. No. 927-60001). To examine catalase protein abundance, the PVDF membrane was incubated overnight with a primary antibody targeting the catalase protein (Abcam, Cat. No. ab52477; 1:1000 dilution). To examine protein abundance of mitochondrial electron transport system complexes, the PVDF membranes were incubated overnight with an antibody cocktail targeting protein subunit of each electron transport system complex (Abcam, Cat. No. ab110413; 1:1000 dilution). The OXPHOS antibodies were validated using isolated mitochondria as a control supplied by Abcam. The following morning, membranes were washed with tris-buffered saline containing 0.1% (v/v) Tween-20 and then incubated with appropriate secondary antibodies conjugated to IRDye 680RD or 800CW from Licor (1:15,000 dilution). Images were acquired using a Licor Odyssey DLx.

### In-situ tibialis anterior (TA) muscle function testing

The contractile properties of the TA muscle were determined using the Aurora Scientific in-situ muscle testing system as previously described^37^. Briefly, mice were anesthetized by an intraperitoneal injection of ketamine (90 mg/kg) and xylazine (10 mg/kg) after which a small portion of skin on top of the dorsal foot was removed to expose the distal tendon of the TA muscle. A double square knot was positioned distal to the myotendinous junction of the tendon of the TA muscle using 4-0 silk suture. A double square knot using 4-0 silk suture was positioned on the patella tendon and the left sciatic nerve was carefully exposed and separated from the surrounding fascia. Thereafter, the animal was moved to the prewarmed testing platform, and the knee was secured using the patella ligament suture lines and foot with adhesive tape. Next, the suture on the distal TA tendon was attached to the lever arm and small hook electrodes were placed under the sciatic nerve and covered by a drop of mineral oil. Optimal length for active force was found using twitch (1 Hz) stimulation at increasing lengths with 30-s intervals between stimuli. After obtaining optimal length, the contractile function was tested using a series of isometric contractions (1, 25, 50, 75, 100, 125, 150, and 175 Hz, 0.2-ms pulses for 300 ms) with 60 s intervals between stimuli (at supramaximal voltage).

### Immunofluorescence microscopy

Skeletal myofiber cross-sectional area (CSA) and capillary density were evaluated using immunofluorescence microscopy as previously described^23^. Following the in-situ muscle functional testing, the TA, EDL, and soleus muscles were dissected, placed in disposable base molds with embedding medium compound, and frozen in liquid nitrogen-cooled 2-methylbutane. Three transverse section (10 µm) of each muscle specimen were cut at the mid-belly using a Leica 3050S cryostat at − 20 °C, mounted on microscope slides, stored at − 20 °C until immunofluorescence staining. Frozen muscle sections were briefly air-dried at room temperature and fixed with 4% paraformaldehyde for five minutes. Following five washes with 1× phosphate buffered saline (PBS), sections were permeabilized with 0.3% Triton X-100 in PBS for ten minutes. After five washes with 1× PBS, sections were incubated in blocking buffer (5% goat serum and 1% bovine serum albumin in PBS) for 1 h. Thereafter the sections were incubated with primary antibody against to laminin (Millipore-Sigma, Cat. No. L9393, 1:100 dilution) overnight at 4 °C to label the sarcolemma. After multiple washes with 1× PBS, sections were stained with a secondary antibody in blocking buffer as follows: for mice treated with AAV-HSA-GFP were labeled with Alexa Fluor 488 goat anti-rabbit IgG (1:250, ThermoFisher Scientific, Cat. No. A-11008), whereas mice treated with AAV-HSA-mCAT were labeled with Alexa Fluor 555 goat anti-rabbit IgG (1:250, ThermoFisher Scientific, Cat. No. A-21428), and both were labeled with Dylight 649-conjugated *Griffonia Simplicifolia* Lectin I Isolectin B4 (Vector Laboratories, Cat. No. DL-1208) to visualize capillaries. Coverslips were mounted with fluorescent mounting medium (Vector Laboratories, Cat. No. H-1500). Slides were imaged at × 20 magnification with an Evos FL2 Auto microscope (ThermoFisher Scientific). Myofiber CSA and capillary density were analyzed using MuscleJ^38^ using tiled images of the entire muscle section. A representative image of AAV-HSA-GFP expression in the tibialis anterior muscle was generated by imaging the native GFP signal with counter staining of myofiber membranes with wheat germ agglutinin conjugated to Alexa Fluor 594 (ThermoFisher Scientific, Cat. No. W11262, 5 μg/ml) and coverslip mounted with.

### Assessment of post-synaptic area morphology

Following the in-situ muscle functional test, the TA muscle was removed from the AVF limb and half of the muscle was placed in a Sylgard®-covered petri dish filled with ice cold PBS and the myofibers were gently teased apart using 45-degree angled forceps. Next, the muscles were fixed in 3.2% PFA for 10 min, washed in PBS, and permeabilized with 2% Triton X-100 at room temperature for 30 min before being placed in blocking buffer (PBS supplemented with 1% Triton X-100 and 4% bovine serum albumin) at 4 °C overnight. After blocking, muscle bundles were incubated with Alexa Fluor-488 conjugated alpha-bungarotoxin (ThermoFisher Scientific, Cat. No. B13422) for 24 h to label the post-synaptic acetylcholine receptor clusters. On the next day, muscle bundles were washed in PBS and then mounted on a microscope slide with mounting medium (Vector Laboratories, Cat. No. H-1500) and sealed with hard nail polish. Thereafter, slides were imaged to visualize the post-synaptic acetylcholine cluster using a confocal microscope (Leica DMI 8) with the following acquisition parameters: Objective: 40x, Format: 1024 × 1024 pixels, Speed: 600 Hz, Bidirectional: ON, Z-step size: 0.5 µM. Acquired images (25.9 ± 12.7 myofibers analyzed per mouse) were quantified using Fiji J semi-automatic macro tool^39^. Key determinants such as acetylcholine receptor (AChR), motor endplate, compactness (AChR area/endplate area × 100) and fragmentation (1–1/number of AChR clusters) were quantified. Investigators involved in the imaging and analysis process were blinded to the treatment conditions.

### Isolation of skeletal muscle mitochondria

Mitochondria from the gastrocnemius and plantaris muscles were isolated as previously described^17,34^. Briefly, the plantaris and gastrocnemius muscles were carefully dissected, such that blood, fat, and connective tissues were removed, and the muscles were minced with scissors on an ice-cold petri dish. Thereafter, the minced muscle was incubated in ice-cold mitochondrial isolation media (MIM) (50 mM MOPS, 100 mM KCl, 1 mM EGTA, 5 mM MgSO_4_, pH 7.2) containing 0.025% w/v trypsin for three minutes, followed by centrifugation at 800×*g* for 5 min at 4 °C. The resulting supernatant containing trypsin was decanted and the pellet was resuspended with approximately 12 ml of MIM with bovine serum albumin (BSA 2 g/l). The sample was homogenized on ice using a glass-Teflon homogenizer and centrifuged at 800xG for ten minutes at 4 °C. The resulting supernatant was transferred into a new tube and centrifuged again at 10,000×*g* for 10 min at 4 °C resulting in a mitochondrial rich pellet. The pellet was washed with MIM (without BSA) to remove damaged mitochondria and residual BSA and then gently resuspended in MIM (without BSA). Protein concentration of the resuspension was assessed using bicinchoninic acid protein assay (ThermoFisher Scientific, Cat. No. A53225).

### Assessment of mitochondrial function

High-resolution respirometry using the Oxygraph-2k (O2K) system (Oroboros Instruments, Innsbruck, Austria) was utilized to assess mitochondrial respiratory function as described previously^23,34^. Oxygen flux (*J*O_2_) was measured using an assay that employs a creatine kinase (CK) energetic clamp that facilitates measures of *J*O_2_ across a range of ATP free-energy states from near resting to maximum contractions (mimicking a stress test)^40–42^. Mitochondria were energized by the additions of 10 mM pyruvate, 2 mM malate, and 2.5 mM octanoylcarnitine. The integrity of the outer mitochondrial membrane was assessed by the addition of cytochrome c (5 µM), and samples exhibiting more than a 25% increase in *J*O_2_ were excluded from analysis. The slope of the relationship between *J*O_2_ and the extramitochondrial free energy for ATP hydrolysis (ΔG_ATP_), termed oxidative phosphorylation (OXPHOS) conductance, was calculated. Mitochondrial hydrogen peroxide production (*J*H_2_O_2_) was measured in all mice whose mitochondrial isolation yielded sufficient quantity of mitochondria. In these cases, isolated mitochondria were energized with 10 mM succinate and hydrogen peroxide emission was measured via the Amplex Ultra Red/horseradish peroxidase detection system as previously described^17,43^ using a Quantmaster-400 (Horiba Instruments) with Excitation = 565 nm and Emission = 590 nm. Fluorescence values were converted to pmoles of H_2_O_2_ using a standard curve.

### Statistical analysis

Data are presented as mean ± SD. Normality of data was tested with the Shapiro–Wilk test. Comparisons of data with more than two groups or with repeated measurements were performed using two-way or three-way ANOVA with Šidák’s post-hoc testing for multiple comparisons when significant interactions were detected. All statistical analysis was performed in GraphPad Prism (Version 9.0). In all cases, *P* < 0.05 was considered statistically significant.

## Results

**Figure 1:**
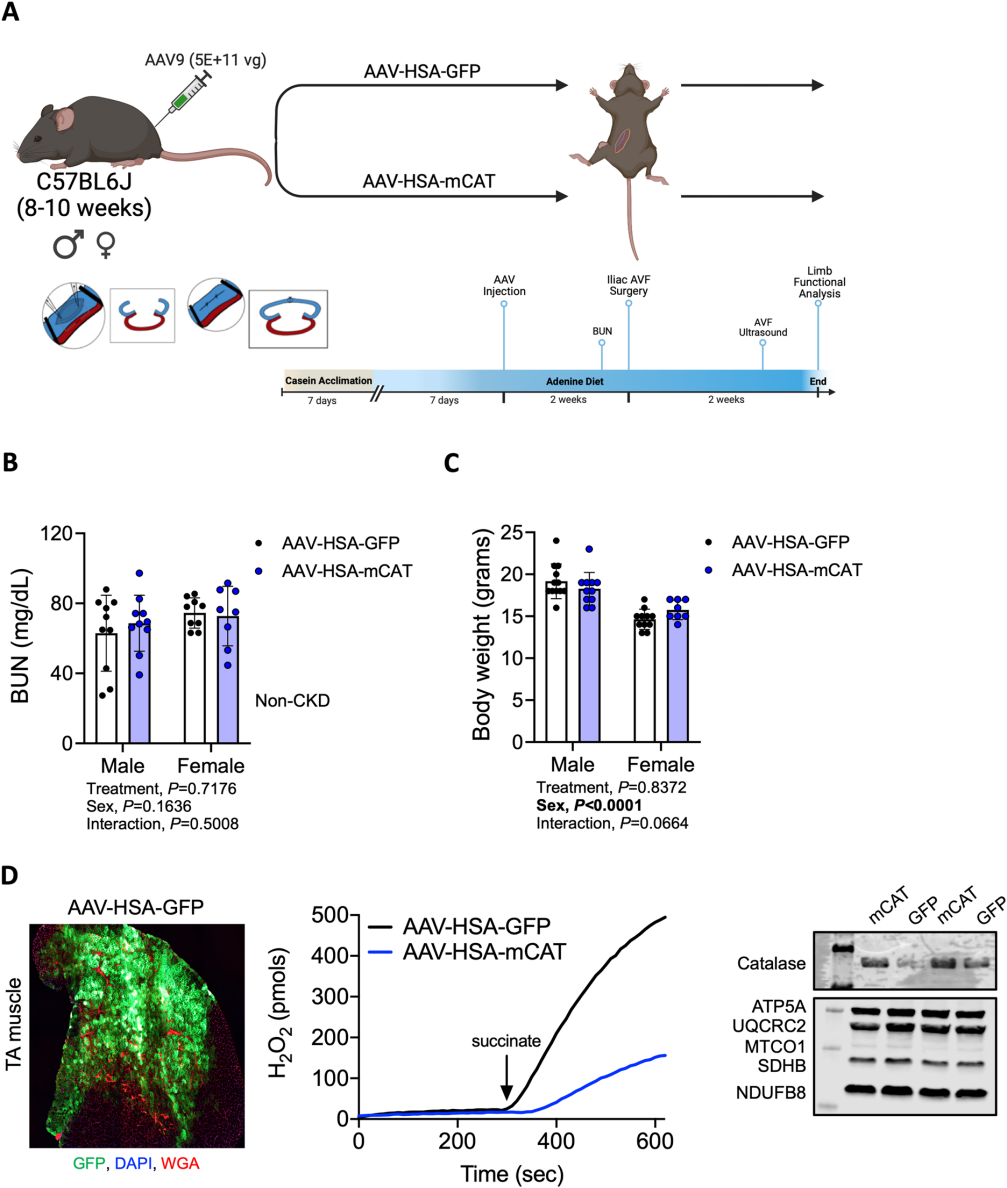
Experimental overview and verification of mouse models and treatments. (**A**) Graphical depiction of the study design. (**B**) Blood urea nitrogen level following 2 weeks of 0.2% adenine supplemented diet (N = 8–10/group/sex). Dashed line represents average value of BUN from animals without CKD. (**C**) Body mass measured at 14 days after the AVF surgery (N = 8–10/group/sex). (**D**) Representative immunofluorescence image of the tibialis anterior (TA) muscle of a mouse treated with AAV-HSA-GFP showing robust expression of the GFP. Mitochondrial H_2_O_2_ emission in mitochondria from AAV-HSA-GFP and AAV-HSA-mCAT mice validating greater mitochondrial H_2_O_2_ scavenging caused by the mCAT. Western blot verification of increased catalase expression in muscle. Data were analyzed using a two-way ANOVA with Šidák’s post-hoc testing. Values are mean ± SD.

To explore if mitochondrial-targeted catalase expression prior to AVF creation could mitigate limb pathophysiology, we randomized mice to receive intramuscular injections of either AAV-HSA-GFP or AAV-HSA-mCAT. Since all patients receiving an AVF have chronic renal insufficiency, mice used in this study had CKD induced by adenine feeding to mimic the metabolic state induced by uremia. A graphical display of the experimental timeline is shown in Fig. 1A. Blood urea nitrogen (BUN) levels, assessed three days before the AVF surgery, were higher in both male and female mice fed the 0.2% adenine diet compared to animals without CKD (dotted line is the average value of plasma BUN from non-CKD mice from our previous study^44^, but was not impacted by muscle-specific expression of either the GFP or mCAT (Treatment effect, *P* = 0.72, Fig. 1B). Body weight was also unaffected by AAV treatments (*P* = 0.84), but females were significantly smaller than males as expected (*P* < 0.001, Fig. 1C). To verify that AAV treatment was successful, immunofluorescence imaging of the TA muscle confirmed robust infection with AAV-HSA-GFP (Fig. 1D). Additionally, we validated successful mCAT expression by measuring succinate-supported mitochondrial H_2_O_2_ emission, which was significantly lower in mice treated with AAV-HSA-mCAT (*P* = 0.0004, Figure 1D). Overexpression of catalase was also substantiated by immunoblotting for catalase protein abundance in AAV infected muscles (Fig. 1D).

### Muscle-specific mCAT expression does not alter vascular hemodynamics or muscle capillary density following AVF surgery

**Figure 2:**
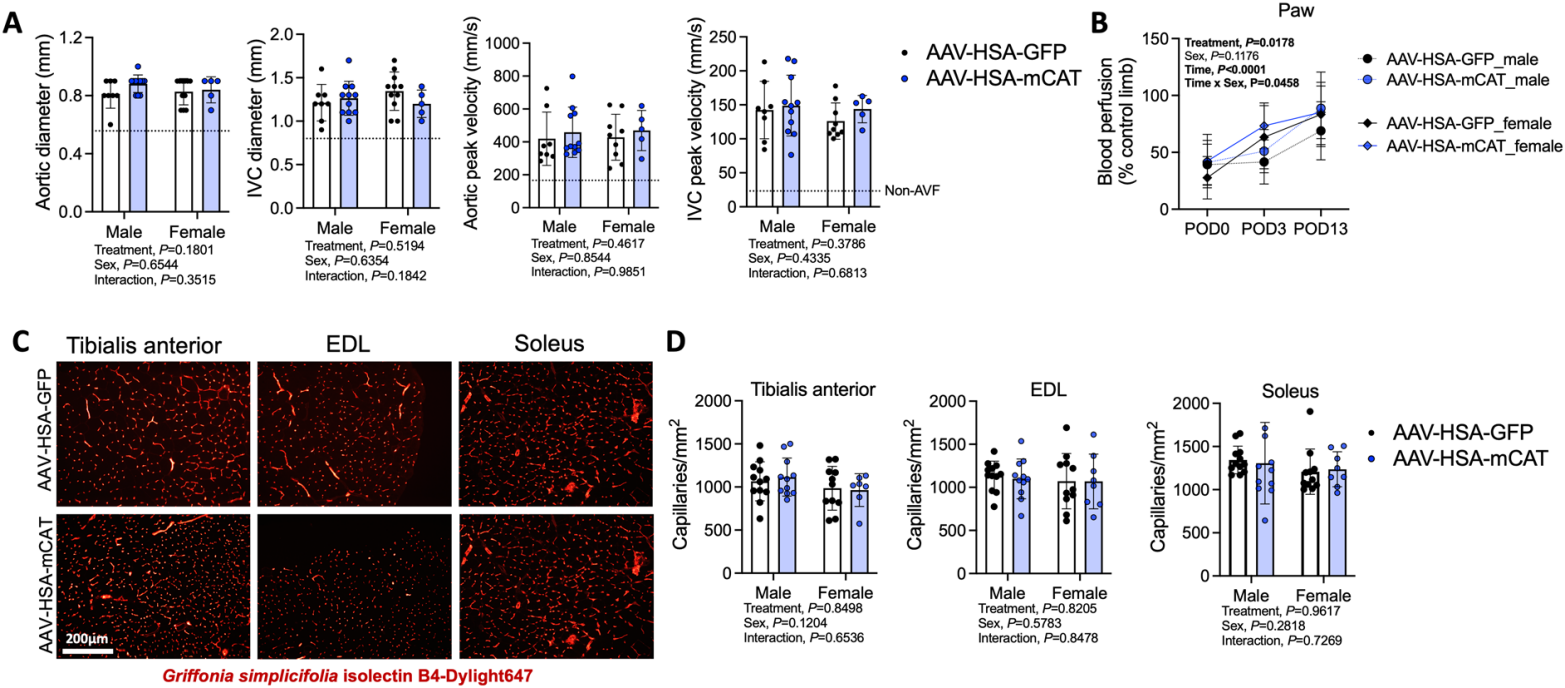
AAV-HSA-mCAT expression does not impact central and peripheral hemodynamics or hindlimb muscle capillarization following AVF surgery. (**A**) Doppler ultrasound assessment of systemic hemodynamics captured at infrarenal aorta and inferior vena cava at POD10 (N = 5–10/group/sex). Dotted lines represent vessel diameters and flow velocities in mice without an AVF from our previous study^23^. (**B**) Blood perfusion of the ventral paw immediately post-operatively, and at POD3 and POD13 (N = 8–10/group/sex). (**C**) Representative images of muscle labeled for capillaries using *Griffonia Simplicifolia* isolectin. Images were from male mice. (**D**) Capillary density of tibialis anterior, EDL, and soleus muscles (N = 8–10/group/sex). Data in panel (**B**) were analyzed using mixed-effects analysis. All other data were analyzed using a two-way ANOVA with Šidák’s post-hoc testing. Values are mean ± SD.

Doppler ultrasound assessments of the infrarenal aorta and inferior vena cava confirmed AVF patency postoperatively (Fig. 2A) as we have previously reported^18,19,23^. As expected, AAV-HSA-mCAT treatment did not impact the central hemodynamics (Fig. 2A). Creation of the arteriovenous anastomosis was associated with an ~ 60% decrease in paw perfusion which gradually recovered across the 2-week post-operative period in all groups. Significant main effects of time (*P* < 0.001) and treatment (*P* = 0.0178) were detected; however, post-hoc testing did not uncover significant effects of treatment in either male (*P* = 0.1528) or female (*P* = 0.3381) mice at POD13 (Fig. 2B). Notably, a time by sex interaction (*P* = 0.0458) was observed indicating that female mice had better paw perfusion recovery compared to male mice. Next, we measured the capillary density within the tibialis anterior, EDL, and soleus muscles harvested 14 days after AVF creation (Fig. 2C,D). In each of the muscles, no significant effects of either treatment or sex were detected (Fig. 2D).

### Muscle-specific mCAT expression does not impact hindlimb muscle mass but improves contractile function following AVF creation

**Figure 3:**
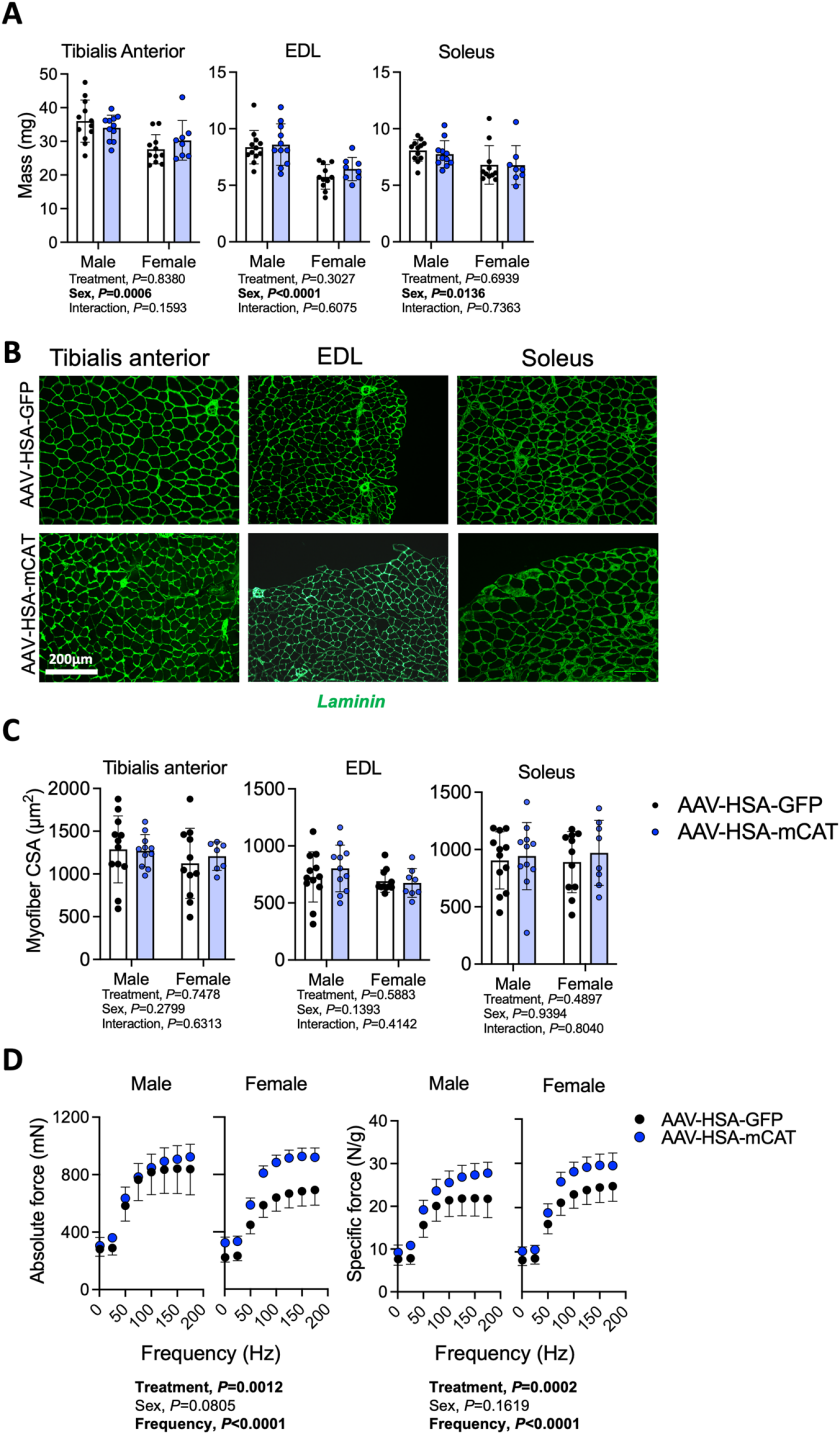
AAV-HSA-mCAT expression does not impact muscle mass or myofiber area, but improves muscle contractile strength following AVF surgery. (**A**) Muscle mass of tibialis anterior, extensor digitorum longus (EDL), and soleus muscles (N = 8–10/group/sex). (**B**) Representative images of muscles immunolabeled for laminin (myofiber membranes). Images were from male mice. (**C**) Mean myofiber cross-sectional area (CSA) in the tibialis anterior, EDL, and soleus muscles (N = 8–10/group/sex). (**D**) In-situ muscle contractile function of TA muscle at POD14 (N = 5–10/group/sex). Data were analyzed using a two-way ANOVA with Šidák’s post-hoc testing. Values are mean ± SD.

Next, we investigated the impact of AAV-HSA-mCAT treatment on hindlimb neuromuscular function and hindlimb muscle pathology. As expected, there were significant sex effects associated with hindlimb muscle mass assessments (Fig. 3A); however, no effect of AAV-HSA-mCAT was detected in any of the muscles (Fig. 3A). Similarly, immunolabeling of the myofiber membrane with laminin revealed that AAV-HSA-mCAT treatment did not significantly impact the mean myofiber cross-sectional area (CSA) within these muscles (Fig. 3B,C). To assess neuromuscular contractile function, we also performed rigorous analyses of isometric muscle contractile function using nerve-mediated stimulation to activate all the motor units within the TA muscle using supramaximal stimulation of the sciatic nerve. Force frequency experiments revealed that mice treated with AAV-HSA-mCAT had significantly *higher* levels of absolute (*P* = 0.0012) and specific force (*P* = 0.0002, absolute force normalized the muscle mass) (Fig. 3D).

### Muscle-specific mCAT expression preserves the post-synaptic neuromuscular junction morphology following AVF creation

**Figure 4:**
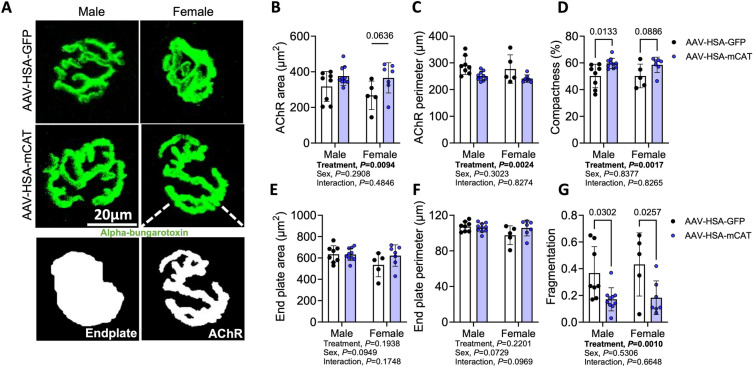
AAV-HSA-mCAT expression preserves the post-synaptic neuromuscular junction morphology following AVF surgery. (**A**) Representative images of the post-synaptic neuromuscular junction from AAV-HSA-GFP and AAV-HSA-mCAT mice. Area of post-synaptic AChR (**B**) and motor endplate (**E**). Perimeter of post-synaptic AChR (**C**) and motor endplate (**F**). (**D**) AChR compactness (AChR area/endplate area × 100). (**G**) Fragmentation (1 − 1/number of AChR clusters). Data were analyzed using a two-way ANOVA with Šidák’s post-hoc testing. Values are mean ± SD. N = 5–10/group/sex.

We next explored whether AAV-HSA-mCAT treatment impacted the morphology of the post-synaptic neuromuscular junction. The acetylcholine receptor (AChR) clusters present on the myofiber membrane were labeled with alpha-bungarotoxin and images were acquired with confocal microscopy. A significant treatment effect was detected for the AChR area (*P* = 0.0094, Fig. 4A,B); however, the AChR perimeter was significantly smaller in AAV-HSA-mCAT treated muscles (*P* = 0.0024, Fig. 4A,C) resulting from a more compact AChR structure (*P* = 0.0017, Fig. 4A,D). No significant effects were detected for the total endplate area or perimeter (Fig. 4E,F) indicating that NAC treatment increased the area of the post-synaptic motor endplate. Strikingly, AAV-HSA-mCAT treated mice displayed significantly *lower* fragmentation of the post-synaptic neuromotor junction (NMJ) compared to AAV-HSA-GFP treated mice following AVF surgery regardless of biological sex (Fig. 4G).

### Muscle-specific mCAT expression does not impact skeletal muscle mitochondrial OXPHOS function following AVF surgery

**Figure 5:**
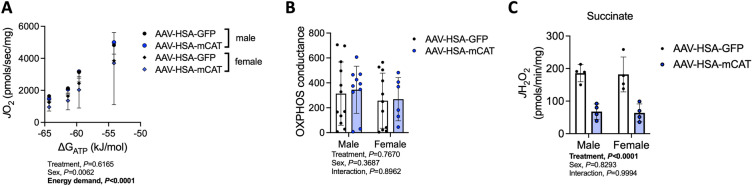
AAV-HSA-mCAT expression reduces mitochondrial hydrogen peroxide emission but does not impact mitochondrial OXPHOS following AVF surgery. (**A**) Rate of oxygen consumption (*J*O_2_) of mitochondria isolated from the plantaris and gastrocnemius muscles measured at various energy demands mimicking the stress test from near resting condition to the maximum contractions. (**B**) Conductance of oxidative phosphorylation (OXPHOS) calculated from the slope of the relationship between *J*O_2_ and energy demand (ΔG_ATP_). (N = 6–12/group/sex). (**C**) Rate of hydrogen peroxide production (*J*H_2_O_2_) determined in isolated mitochondria fueled by 10 mM succinate (N = 4/group/sex). Data were analyzed using a two-way ANOVA with Šidák’s post-hoc testing. Values are mean ± SD.

Utilizing a recently developed mitochondrial “stress test”^41^, AAV-HSA-mCAT treatment was found to have no effect on mitochondrial respiration rate across a range of physiologically-relevant energy demands (ΔG_ATP_ refers to the free energy of ATP hydrolysis) (*P* = 0.62, Fig. 5A). Quantification of the slope of the relationship between *J*O_2_ (oxygen consumption) and energy demand (ΔG_ATP_), termed OXPHOS conductance, further confirmed that AAV-HSA-mCAT expression has no impact on mitochondrial energy transduction compared to AAV-HSA-mCAT treated counterparts (*P* = 0.77, Fig. 5B). In a subset of samples where the mitochondrial yield was sufficient for parallel analysis of hydrogen peroxide production (*J*H_2_O_2_), AAV-HSA-mCAT was found to significantly *decrease* succinate-supported hydrogen peroxide production (*P* < 0.0001, Fig. 5C), but there were no sex or interaction effects detected (Supplementary Fig. 1).

## Discussion

The present study was designed to investigate the therapeutic impact of mitigating mitochondrial hydrogen peroxide production on access-related limb dysfunction in a recently developed uremic murine iliac AVF model. The basis of this approach stems from the body of literature demonstrating that the CKD promotes oxidative stress in skeletal muscle and such effects have been linked mechanistically to muscle atrophy and contractile dysfunction^11–13,16,28,45–49^. In support of this, we recently demonstrated that systemic *N*-acetylcysteine treatment can attenuate neuromotor dysfunction in this murine iliac AVF model^37^. Considering the systemic nature of *N*-acetylcysteine, we next wanted to test the role of mitochondrial-derived ROS in the etiology of AVF-induced limb dysfunction. To accomplish this, we utilized AAV9 to express catalase fused to a mitochondrial-targeting sequence driven by the human skeletal actin promoter to limit expression only to myofibers^35,50^ and this treatment was applied prior to the creation of an iliac AVF in mice with CKD. The major findings from this study are that AAV-HSA-mCAT treatment resulted in a significant increase in muscle contractile function and preserved the morphology of the post-synaptic neuromuscular junction. Notably, the improve limb muscle functionality occurred without significant changes in hemodynamics, capillary density, or mitochondrial OXPHOS.

While creation of an AVF has emerged as the preferred approach for creating durable vascular access for hemodialysis^4,51,52^ in most patients, there remains a spectrum of hand dysfunction that can be observed following these operations^4,51,53^. In its most severe manifestation, access-related hand ischemia can result in motor disability, rest pain and/or tissue loss which often requires surgical remediation^51,54–56^. However, principal component analysis on a well-phenotyped cohort of ESKD patients receiving AVF surgery reported that post-operative changes in hand function (motor and sensory) were largely independent of the hemodynamic changes^4^. Similarly, Khemtova et al.^53^ reported that decreases in grip strength following AVF creation were detectable without clinically overt hand ischemia, although subtle tissue oxygenation changes may play a role in hand dysfunction. In totality, these clinical observations suggest the possibility that tissue-related characteristics may contribute to the susceptibility of hand dysfunction following AVF creation. In this study, a murine AVF model was used to test the contribution of skeletal muscle to access-related limb pathophysiology. Intriguingly, expression of a mitochondrial targeted catalase specifically in skeletal muscle fibers significantly *improved* muscle strength following AVF creation (Fig. 3D). Importantly, improvements in muscle strength occurred without changes in limb hemodynamics or muscle size/mass supporting the assertion that resident muscle biology contributes to access-related hand dysfunction.

There is a wealth of literature linking excess mitochondrial reactive oxygen species to muscle atrophy and weakness in other conditions such as aging^57–61^, disuse^62^, heart failure^63^, and cancer chemotherapy^64^. Similar to the findings of this study with AVF limb muscle weakness, Xu et al.^58^ reported that expression of mitochondrial targeted catalase mitigated the muscle atrophy and weakness in CuZn superoxide dismutase (*Sod1*) knockout mice. Likewise, overexpression of the mitochondrially-localized peroxiredoxin 3 (*Prdx3*) has also been reported to preserve muscle weakness in *Sod1* knockout mice^65^. Mitochondrial-targeted catalase has also been reported to preserve muscle contractile function in a breast cancer mouse model coupled with chemotherapy^64^. The underlying mechanisms driving muscle weakness following AVF creation are unknown, but there is evidence that redox-sensitive post-translational modifications to cysteine residues occur in several myofilament proteins (reviewed in Ref.^57^). For example, oxidation of proteins involved in excitation–contraction coupling, such as the ryanodine receptor^66^ or glutathionylation of the sarco-endoplasmic reticulum calcium ATPase (SERCA)^59^, have been reported to cause muscle weakness. Using redox proteomics in a rat heart failure model that causes diaphragm muscle weakness, Kelley et al.^67^ identified greater cysteine oxidation of thin filament protein including actin, troponin I, and tropomyosin, as well as methionine oxidation of actin and myosin light chains; however a cause-effect relationship between these oxidative modifications and muscle weakness has not been established. In the context of access-related hand dysfunction, more work is needed to elucidate how the complex uremic state coupled with hemodynamic alterations caused by AVF creation impact oxidative modifications to muscle contractile proteins. Nonetheless, there is evidence that the uremic state present in ESKD is associated with protein oxidation in skeletal muscle^25,26^, suggesting that redox modification of muscle proteins could partially explain the wide spectrum of ARHD that is observed following AVF creation.

There are some limitations of the current study worthy of discussion. First and foremost, mice with CKD in this study were not subjected to hemodialysis treatments following creation of the AVF and long-term AVF maturation is unlikely to have occurred considering the short post-operative period. In contrast to this, it is relatively common among the subset of ESKD patients that receive AV-access surgery to receive catheter-based hemodialysis post-AVF surgery while maturation occurs prior to the newly placed access being converted to the primary vascular access. The impact of catheter-based dialysis during the post-operative period on the development of access-related hand dysfunction is unknown. Second, the experimental design was relatively short in this investigation with terminal measures of limb function performed 2 weeks after AVF creation which is much shorter than the timeframe in CKD and ESKD patients undergoing autogenous AVF placement where the access is typically allowed to mature for 6 weeks or longer before being used for hemodialysis access. Third, CKD and ESKD patients often present with numerous additional co-morbid conditions (e.g., diabetes, hypertension, hyperlipidemia, etc.) and are taking multiple medications to manage these problems to mitigate cardiovascular disease risk. The impact of these different co-morbid conditions and associated medication exposure on the neuromuscular system are unknown and were not present in mice with adenine-induced CKD that were employed in this study.

In conclusion, decreasing mitochondrial ROS levels via muscle-specific expression of a mitochondrial-targeted catalase significantly improved muscle strength and the neuromuscular junction morphology following AVF surgery in mice with CKD. The attenuation of AVF-induced limb dysfunction occurred without altering central or peripheral hemodynamics or muscle size, implicating muscle oxidative stress as a key determinant of access-related hand dysfunction. Future clinical studies are needed to explore muscle pathophysiology in CKD/ESKD patients undergoing autogenous AVF placement as exogenous antioxidants therapies may provide a novel preventative treatment option to improve hand outcomes in this population.

### Supplementary Information

Supplementary Information.

## Data Availability

The datasets used and/or analyzed during the current study available from the corresponding author on request.
