# Supplementary material for: Mitochondrial targeted catalase improves muscle strength following arteriovenous fistula creation in mice with chronic kidney disease
Source: Sci Rep. 2024 Apr 9;14:8288. doi: 10.1038/s41598-024-58805-1 (PMC11004135; doi:10.1038/s41598-024-58805-1)
Supplement: Supplementary file 1 — Supplementary Information. [file 41598_2024_58805_MOESM1_ESM.pdf]

SUPPLEMENTAL MATERIAL

Mitochondrial Targeted Catalase Improves Muscle Strength Following Arteriovenous Fistula Creation in Mice with Chronic Kidney Disease

Kyoungrae Kim[2], Brian Fazzone[1,4], Tomas A. Cort[2], Eric M. Kunz[2], Samuel Alvarez[2], Jack Moerschel[2], Victoria R. Palzkill[2], Gengfu Dong[2], Erik M. Anderson[1,4], Kerri A. O'Malley[1,4], Scott A. Berceli[1,4], Terence E. Ryan[2,3#], Salvatore T. Scali[1,4#]

[1]Division of Vascular Surgery and Endovascular Therapy, [2]Department of Applied Physiology and Kinesiology, [3]Center for Exercise Science, University of Florida, Gainesville, FL. [4]Malcom Randall Veteran Affairs Medical Center, Gainesville, FL.

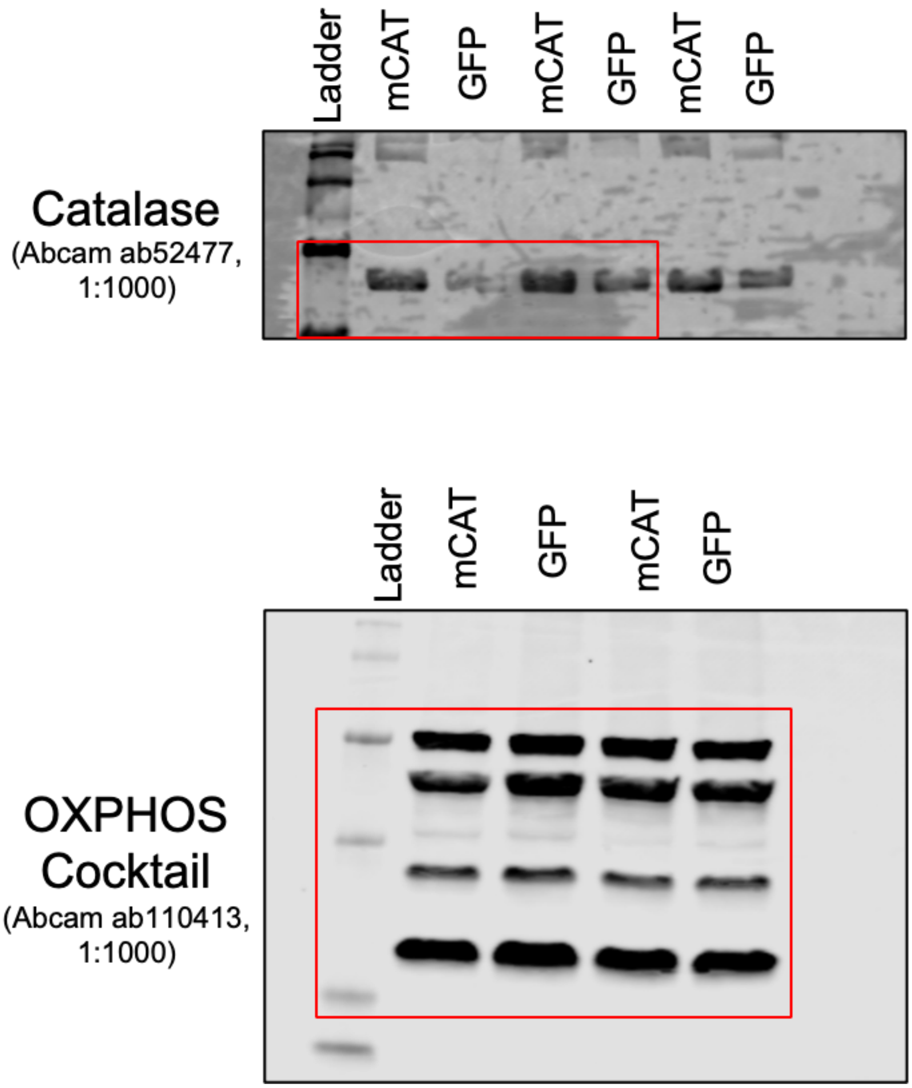

Supplemental Figure 1: Uncropped western blots related to Figure 1D.

Below are the raw imaging files and experimental properties for obtain the images via the Licor Imager.

OXPHOS Cocktail

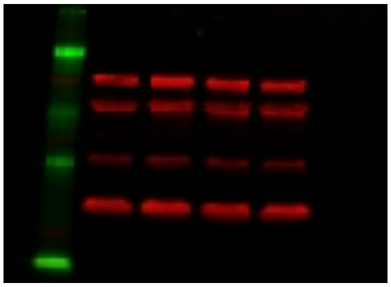

#Properties Updated
#Tue Nov 26 11:29:06 EST 2019
MouseStrain=
Y=23.0
X=24.0
BackgroundSegments=All
CropCenterPt=165, 121
FileVersion=ACQ VERSION 2.0
Quality=high
Barcode=
AnimalId=
FlipImage=false
Treatment=
OrganizationName=
Intensities=Auto Auto
CropDimension=310, 222
ConcentrationColumn=Signal
TimeStamp=2019, 10, 26, 11, 20, 49, 809
InstrumentModel=Odyssey CLx
AcqResolution=169um
ImageModifications=
BackgroundMethod=Median
CropAnnotationColor=BlueYellow
ExRunID=
700_ChannelSettings=500, 20067, 23548, 0, 0, 0, 0
Archived=false
InstrumentName=CLX-1239
InstrumentVersion=1.0.11
Experiment=
Orientation=
LastUsedTextId=0
Height=41.0
ImageName=0006025_01
AnalysisType=None
AcquireTimeZoneId=America/New_York
ProjInfo=
ImageId=0006025_01
InjectTime=
UserInfo=
FocusPosition=0.0

CustomField2=
CustomField1=
CustomField0=
Agent=
FileType=LI-COR Image Studio Acquisition File
InterpolationMethod=Linear
ChannelFileNames=0006025_01_700.TIF, 0006025_01_800.TIF
CellLine=
Width=56.0
TimePoint=1900, 0, 1, 0, 0, 0, 0
ChannelNames=700, 800
NormalizationChannel=
ChannelPreviews=false, false
BackgroundWidth=3
ProjectName=
LastUsedShapeId=0
800_ChannelSettings=550, 16709, 20718, 0, 0, 0, 0
Remarks=

<u>Catalase</u>

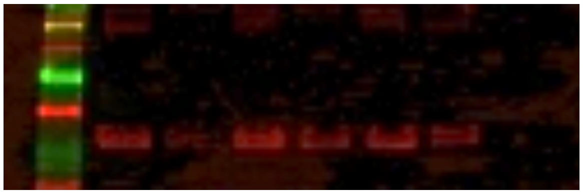

#Properties Updated
#Tue Nov 26 11:41:13 EST 2019
MouseStrain=
Y=40.0
X=15.0
BackgroundSegments=All
CropCenterPt=200, 65
FileVersion=ACQ VERSION 2.0
Quality=high
Barcode=
AnimalId=
FlipImage=false
Treatment=
OrganizationName=
Intensities=Auto Auto
CropDimension=381, 110
ConcentrationColumn=Signal
TimeStamp=2019, 10, 26, 11, 35, 55, 742
InstrumentModel=Odyssey CLx
AcqResolution=169um
ImageModifications=
BackgroundMethod=Median

```
CropAnnotationColor=BlueYellow
ExRunID=
700_ChannelSettings=500, 18351, 21449, 5000, 0, 0, 0
Archived=false
InstrumentName=CLX-1239
InstrumentVersion=1.0.11
Experiment=
Orientation=
LastUsedTextId=0
Height=22.0
ImageName=0006028_01
AnalysisType=None
AcquireTimeZoneId=America/New_York
ProjInfo=
ImageId=0006028_01
InjectTime=
UserInfo=
FocusPosition=0.0
CustomField2=
CustomField1=
CustomField0=
Agent=
FileType=LI-COR Image Studio Acquisition File
InterpolationMethod=Linear
ChannelFileNames=0006028_01_700.TIF, 0006028_01_800.TIF
CellLine=
Width=68.0
TimePoint=1900, 0, 1, 0, 0, 0, 0
ChannelNames=700, 800
NormalizationChannel=
ChannelPreviews=false, false
BackgroundWidth=3
ProjectName=
LastUsedShapeId=0
800_ChannelSettings=550, 16622, 22493, 5000, 0, 0, 0
Remarks=
```
